# The Effect of Yoga and Gastro-Hepatic Pack on Type 2 Diabetes Mellitus: A Four-Arm Randomized Controlled Study

**DOI:** 10.7759/cureus.100378

**Published:** 2025-12-29

**Authors:** Preety Laimujam, Ganesh Prasad, Shivaprasad Shetty, Prashanth Shetty

**Affiliations:** 1 Yoga, Sri Dharmasthala Manjunatheshwara (SDM) College of Naturopathy and Yogic Sciences, Ujire, IND; 2 Fasting Therapeutics, Sri Dharmasthala Manjunatheshwara (SDM) College of Naturopathy and Yogic Sciences, Ujire, IND

**Keywords:** diabetes distress, diabetes mellitus type 2, fasting blood sugar, gastro-hepatic pack, glycated hemoglobin (hba1c), perceived stress scale 10, post prandial blood sugar, sudarshan kriya yoga, yoga therapy

## Abstract

Background: Diabetes mellitus is a leading cause of global mortality with significant health complications. Yoga and naturopathy have demonstrated beneficial effects on blood glucose control and psychological well-being.

Methods: A four-arm randomized study was conducted with 120 type 2 diabetic patients aged 40-60 years. Participants were randomly assigned to YG (yoga, 60 minutes), GH (gastro-hepatic pack, 20 minutes), CYG (combined yoga and gastro-hepatic pack, 80 minutes), or the control group. HbA1c, fasting and postprandial blood sugar, Perceived Stress Scale, and Diabetes Distress Scale were measured before and after intervention.

Result: A significant reduction in glycemic parameters was observed in all groups post-intervention, with HbA1c decreasing from 8.17 ± 1.11 to 6.95 ± 0.85 in YG, 8.04 ± 0.98 to 6.93 ± 0.67 in GH, and 8.23 ± 1.23 to 6.29 ± 0.59 in CYG (p < 0.001). Fasting blood sugar (FBS) and post-prandial blood sugar (PPBS) also significantly declined (p < 0.001). Psychological measures improved, with PSS scores dropping from 17.7 ± 3.2 to 9.80 ± 1.67 in YG and 16.0 ± 2.3 to 9.53 ± 1.48 in CYG; smaller reductions occurred in GH and Control. DDS scores significantly decreased (p < 0.001). CYG showed the greatest overall improvement. No adverse events were reported.

Conclusion: The combined yoga and gastro‑hepatic pack (CYG) intervention outperformed either alone in reducing stress and improving glycemic control in type 2 diabetes. This safe, holistic approach enhances overall quality of life, though long-term studies are needed to confirm the sustainability of these benefits and their impact on diabetes-related complications.

## Introduction

Type 2 diabetes mellitus (T2DM) is one of the most prevalent metabolic diseases in the world, primarily caused by a combination of two major factors: the pancreatic β-cells' impaired ability to secrete insulin and the tissues’ incapacity to react to insulin [1]. The International Diabetes Federation (IDF) reports that 463 million persons between the ages of 20 and 79 had diabetes in 2019, and that number is expected to increase to 700 million by 2045. Diabetes also caused 4.2 million deaths in 2019 [2]. Numerous risk factors, such as age, ethnicity, family history, poor socioeconomic level, obesity, metabolic syndrome, and specific unhealthy lifestyle choices, are linked to the pathophysiology of type 2 diabetes [3].

Long-term stress and obesity do, in fact, produce a vicious cycle that leads to metabolic malfunction. The outcome of this metabolic malfunction is the development of insulin resistance. The sympathoadrenal system and the hypothalamic-pituitary-adrenal (HPA) axis play a key role in modulating the stress response. Insulin resistance and stress hyperglycemia are examples of evolutionarily maintained responses that are produced to help the host survive times of intense stress [3]. Diabetes distress (DD) “refers to the negative emotional or affective experiences resulting from the challenge of living with the demands of diabetes, regardless of the type of diabetes” [4]. Patients with type 2 diabetes are more likely to experience several short- and long-term problems. The problems include malignancies, microvascular illnesses (retinopathy, nephropathy, and neuropathy), and macrovascular disorders (hypertension, hyperlipidemia, heart attacks, coronary artery disease, strokes, cerebral vascular disease, and peripheral vascular disease) [5]. The main goals of the conventional treatment for type 2 diabetes are blood sugar regulation by insulin therapy, oral medicines, and lifestyle changes. Sulfonylureas, metformin, and more recent medications like SGLT2 inhibitors and GLP-1 receptor agonists are frequently used medications. Even while these therapies work, they frequently have drawbacks, including adverse effects, a gradual loss of effectiveness, and trouble achieving ideal glycemic control. A nation’s economy is negatively impacted by widespread drug use, particularly in developing countries like India. The creation of healthier, more practical substitutes is therefore necessary [6]. Medication-assisted diabetes treatment has been created to maintain anthropometric measurements, stress levels, blood glucose, and lipid profiles while modifying lifestyle and behavior [7]. Combining good eating and physical activity with the prescribed dosage of medications for each diabetic patient is one example of a lifestyle intervention. Maintaining physical activity is crucial for general health, blood glucose control in diabetics, and avoiding prediabetes from progressing to type 2 diabetes in high-risk patients. A lifestyle intervention that balances energy use and gain in order to encourage people to sustain their bodily energy. One of the main treatments for this is lowering energy consumption and increasing physical activity [8]. Hence, in recent years, several studies have been conducted to evaluate the effect of an increase in physical activity on managing T2DM and also to prevent its complications.

Yoga is a globally recognized lifestyle method for health and wellness that has been practiced for several centuries in India. Numerous lifestyle disorders, including diabetes, have been found to benefit from yoga. Asanas, or yoga postures and poses, and pranayama, or the practice of regulating one's breath through yoga techniques and exercises, have been shown to improve respiratory, neurological, and cardiometabolic parameters in individuals with diabetes in addition to positively influencing oxidative stress, insulin resistance, and glycaemia [9]. In order to regulate the autonomic nervous system and treat psychological and stress-related disorders, Art of Living (AOL), India, created the unique breathing technique known as Sudarshan Kriya Yoga (SKY) [10]. Gastro-hepatic pack (GHP) is a complementary naturopathic treatment used in managing type 2 diabetes mellitus (T2DM), involving the application of a hot fermentation bag on the abdomen (liver area) and a cold bag on the lower back. Studies indicate that a 20-minute session of GHP can significantly reduce blood glucose levels immediately and up to 15 minutes post-treatment in T2DM patients, suggesting it may be effective as an adjunct therapy for blood glucose management. Additionally, repeated GHP treatment over about a week has shown improvements in liver function parameters, which are often compromised in T2DM. The mechanism is believed to involve increased blood circulation and nutrient supply to abdominal organs due to alternating heat and cold application, enhancing their function and glucose metabolism. Although research is still limited and mostly pilot studies, initial evidence positions GHP as a promising non-pharmacological approach in T2DM care, potentially aiding glycemic control and liver health alongside standard treatments [11].

This study will be conducted to check the effectiveness of yoga and GHP in improving DD, perceived stress, and glucose levels. This is the first four-arm study conducted to evaluate the efficacy of yoga, GHP, combined, and standard care on biochemical and psychological parameters.

## Materials and methods

Design

This was a randomized, open-label, four-arm study.

Participants

**Figure 1:**
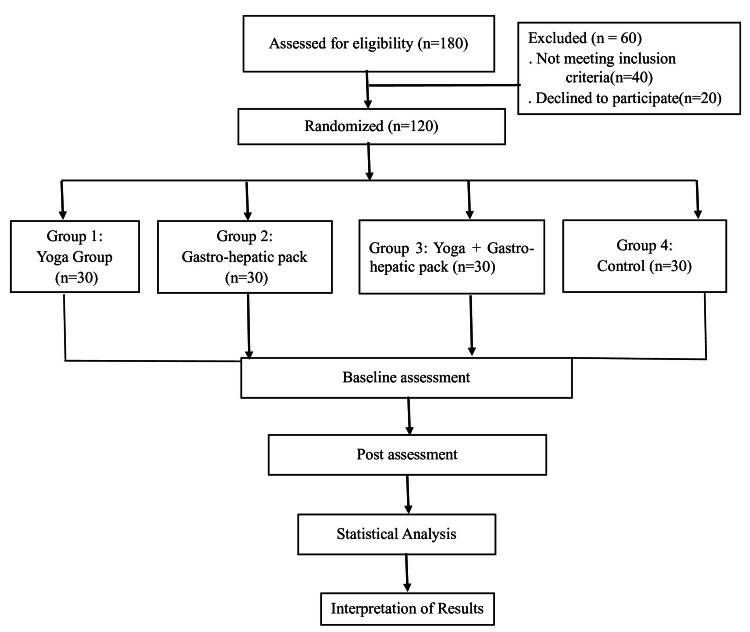
Schematic representation of the study design and sample allocation across groups.

A total of 120 type 2 diabetic patients were recruited from the outpatient unit of yoga, Sri Dharmasthala Manjunatheshwara (SDM) Yoga and Nature Cure Hospital, Ujire, Karnataka. Participants who fulfilled the inclusion criteria were randomly allocated into four groups, with 30 subjects in each group, as explained in Figure 1. A computer-generated random sequence was used for the randomization process.

The study was conducted between October 2024 and September 2025. The clinical protocol was approved by the Institutional Ethics Committee (Ref. no. EC-612) and registered with the Clinical Registry of India. Participants were fully informed about the study, and written informed consent was obtained from each patient prior to the intervention. Assessments were conducted both before and immediately after the intervention sessions. All participants had been on stable oral anti-diabetic therapy for at least one year prior to enrollment. Medication regimens were not altered during the study, and participants continued their usual diet without any imposed dietary restrictions. The variation in the type of oral anti-diabetic medications, including metformin and other oral hypoglycemic agents, reflects routine clinical practice.

Inclusion criteria

Age between 40 and 60 years [12]. Gender- Both Male and female subjects, meeting the American Diabetes Association Diagnostic Criteria, and those who are on oral anti-diabetic therapy, those who are already diagnosed, at least 1 year, and subjects who are willing to participate in the study by signing an informed consent. According to the American Diabetes Association, FBG ≥ 126 mg/dL, 2-h PG ≥ 200 mg/dL, and HbA1c ≥ 6.5 [13].

Exclusion criteria

Type I or gestational diabetes, Participants who are insulin-dependent, participants who had recently undergone surgery, pregnancy, or had cardiac, renal, and proliferative renal complications [14-15].

Assessment

Before the intervention, all the participants underwent a baseline assessment including demographic details, medical history, and clinical evaluation to confirm eligibility. HbA1c: Measured by high-performance liquid chromatography (HPLC) to assess long-term glycemic control. Fasting Blood Sugar and Postprandial: Measured using the glucose oxidase-peroxidase method after an overnight fast and two hours post-meal, respectively. All samples were collected and analyzed in the same certified laboratory to ensure consistency. Perceived Stress Scale (PSS-10): Assessed general psychological stress over the past month (Appendix A) [16]. Diabetes Distress Scale (DDS-17): Evaluated emotional and psychosocial distress related to diabetes (Appendix B). All assessments were conducted pre- and post-intervention to evaluate the impact of the intervention on both metabolic and psychological outcomes [17].

Intervention

The participants selected for the study were randomly assigned to Group 1 (YG), Group 2 (GHG), Group 3 (CYG), and Group 4 (Control).

YG (Yoga Group)

For the first two weeks, the yoga intervention was offered five days a week in the outpatient department, with a 60-minute session each day. Online follow-up yoga sessions were conducted for the remaining 10 weeks to support and continue the practice. During this period, participants practiced four days a week. Attendance was recorded, with 90% of participants attending regularly, while some attended inconsistently, but none dropped out. All yoga sessions were conducted in the morning (6-7 AM) on an empty stomach. And yoga was taught by the same instructor throughout the session.

**Table 1:** Yoga Protocol Asanas [18], Sudarshan Kriya Yoga (SKY) [19]

Name of the Practice	Details	Duration
Asanas	Shalabhasana, Bhujangasana, Dhanurasana, Naukasana, Pawanamuktasana, Ardhamatsyendrasana, Parvatasana, Nadishodhana Pranayama, and Bhastrika	20 min
Sudarshan Kriya Yoga (SKY)	Ujjayi or “Victorious Breath”: Involves experiencing the conscious sensation of the breath touching the throat. This slow breath technique (2–4 breaths per minute) increases airway resistance during inspiration and controls airflow so each phase of the breath cycle can be prolonged to an exact count. During Bhastrika or “Bellows Breath,” air is rapidly inhaled and forcefully exhaled at a rate of 30 breaths per minute, causing excitement followed by calmness. “Om” is chanted three times with prolonged expiration.	40 min

GHG (Gastro-Hepatic Pack Group)

A GHP was given in the morning for two weeks and administered every day. Over the following 10 weeks, follow-up appointments were scheduled every three weeks. During each 3-week period, a 10-day treatment will be provided, and the remaining days will be continued at home. Throughout the duration of the treatment, this regimen ensured that the patient’s development was continuously monitored and evaluated. The treatment was administered in a supine lying position, where the hot fomentation bag was kept over the abdominal region, and then the ice bag on the lower back region. Then covered with a cotton cloth and wrapped with a woolen blanket. The duration of treatment was 20 minutes. The application of heat to the abdomen causes increased peripheral circulation and a significant increase in the muscle tissue total hemoglobin level and muscle tissue oxygen saturation in the vicinity of the sheet application [20].

CYG (Yoga and Gastro-Hepatic Pack)

Group 3 underwent both Yoga and Gastro-hepatic pack. Following the first 60-minute yoga session, participants received a GHP treatment every day for two weeks for 20 minutes. Yoga follow-ups were done online for the next 10 weeks, and the GHP follow-ups were scheduled every three weeks. Throughout the study period, this systematic routine made it possible to continuously monitor and integrate both activities.

Control

The Control group continued their standard pharmacological therapy throughout the study period, without any additional intervention. Participants were instructed to follow their prescribed medication regimens carefully, and no changes in type or dosage were allowed during the study to minimize potential confounding effects.

Statistical analysis

The statistical analysis was performed by using Jamovi (2.6.44 version). Data were expressed as mean ± standard deviation (SD). Normality was assessed using the Shapiro-Wilk test. Within-group analyses were conducted using the paired t-test and Wilcoxon signed-ranked test, while between-group analyses were performed using the One-way ANOVA and Kruskal-Wallis. A p-value of 0.05 was considered statistically significant.

## Results

Baseline demographic

The study included a total of 120 participants divided into four groups of 30 each: YG, GH, CYG, and control. The mean age (± SD) for the groups was 49 ± 7.38, 49.9 ± 5.68, 49.8 ± 5.22, and 47.2 ± 5.48 years, respectively. Gender distribution was balanced with slight variations across groups: YG had 12 males (40%) and 18 females (60%), GH had 15 males (50%) and 15 females (50%), CYG had 18 males (60%) and 12 females (40%), and Control had 14 males (46.7%) and 16 females (53.3%). A Chi-square test was performed to compare categorical variables across the four study groups. There was no significant difference in gender distribution among the groups (χ²(3) = 17.8, p > 0.05). These demographic characteristics demonstrate comparability across the study groups (Table 2). Adherence to metformin was observed in 12 subjects (40%) in YG, 16 subjects (53.3%) in GH, 9 subjects (30%), and the control group, 26 subjects (86.6%). The portion of participants receiving concomitant antihypertensive medications was 8 (26.67%) in YG, GH10 (33.3%), CYG 12 (40%), and Control 15 (50%). Pre- and post-intervention means are shown for YG, GHP (GH), combined YG + GH, and Control groups. All intervention groups showed improvement, while the Control group showed a minimal change.

**Table 2:** Baseline demographic characteristics and medication profile of study participants. Values are presented as mean ± standard deviation or n (%). All participants were on oral anti-diabetic therapy at baseline, in accordance with the inclusion criteria. The table presents the proportion of participants receiving metformin and those receiving concomitant antihypertensive medications as part of routine clinical care. No changes in medication type or dosage were made during the study period, and no dietary restrictions were imposed. The χ² test was used for comparison of categorical variables. YG: Yoga group; GH: gastro-hepatic pack group; CYG: combined yoga and gastro-hepatic pack group; χ²: Chi-square

	Age	Males	Females	N	χ²	
YG	49 ± 7.38	12 (40%)	18 (60%)	30	17.8	
GH	49.9 ± 5.68	15 (50%)	15 (50%)	30	18.6	
CYG	49.8 ± 5.22	18 (60%)	12 (40%)	30	13.7	
Control	47.2 ± 5.48	14 (46.7%)	16 (53.3%)	30	29.7	
Metformin (oral anti-diabetic)	YG	GH	CYG	Control		
	12 (40%)	16 (53.3%)	9 (30%)	26 (86.67%)		
						Concomitant antihypertensive medication	8 (26.67%)	10 (33.3%)	12 (40%)	15 (50%)	

Table 3 describes the adherence to the Yoga Program. A total of 60 subjects attended yoga daily, except for 3 in YG and 5 in the CYG. These eight subjects did not drop out but attended yoga only 2-3 days per week, missing 1-2 sessions within each 5-day period.

**Table 3:** Adherence to the Yoga Program YG: Yoga group; CYG: combined yoga and gastro-hepatic pack group

	Did not Attend Once in 5 Days	Number of Subjects
YG	1-2	3 (10%)
CYG	1-2	5 (16.67%)

**Figure 2:**
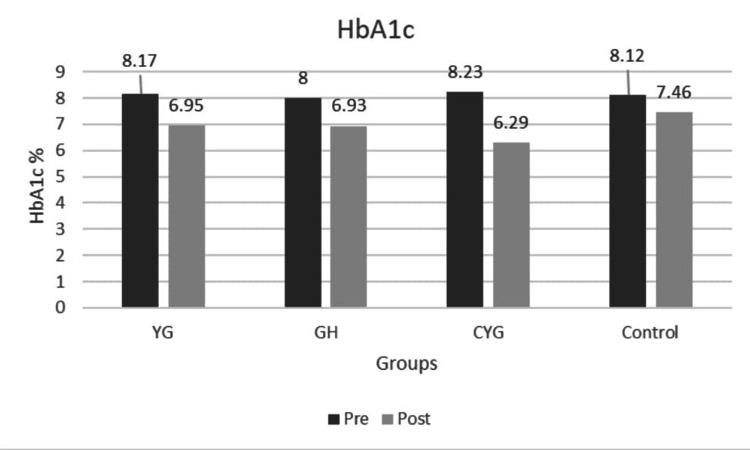
Graph 1: Glycated hemoglobin - Pre and Post Across Groups Pre- and post-intervention mean are shown for Yoga Group (YG), Gastro-hepatic pack (GH), combined YG + GH, and Control groups. All intervention groups showed improvement, while the Control group showed a minimal change.

**Figure 3:**
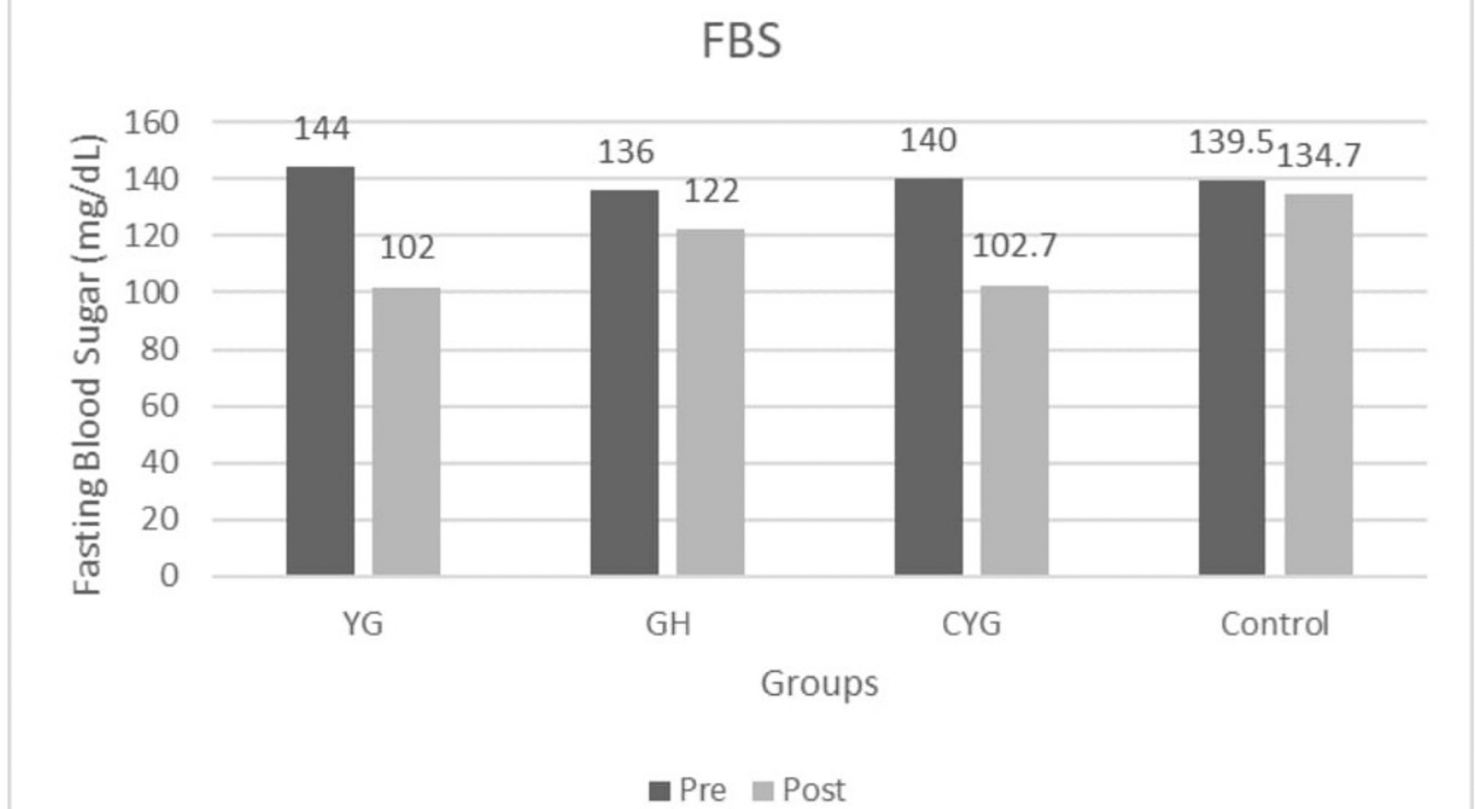
Graph 2: Fasting Blood Sugar - Pre and Post Across Groups Pre- and post-intervention means are shown for Yoga Group (YG), Gastro-hepatic pack (GH), combined YG + GH, and Control groups. All intervention groups showed improvement, while the Control group showed a minimal change.

**Figure 4:**
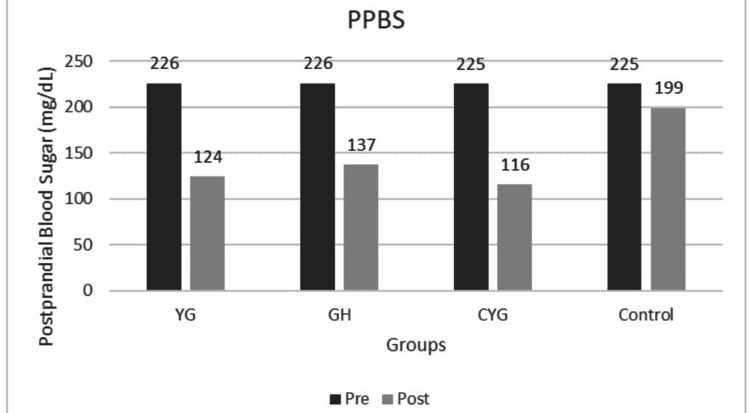
Graph 3: Postprandial Blood Sugar - Pre and Post Across Groups Pre- and post-intervention means are shown for Yoga Group (YG), Gastro-hepatic pack (GH), combined YG + GH, and Control groups. All intervention groups showed improvement, while the Control group showed a minimal change.

The mean HbA1c values decreased substantially from baseline to post-intervention in all intervention groups (YG, GH, CYG), with strong statistical significance (p < 0.001 within groups). The Control group also showed a significant reduction in HbA1c, but to a lesser extent than the intervention groups, and the between-group p-values confirm statistical significance for improved glycemic Control (Figure 2). Fasting blood sugar (FBS) and post-prandial blood sugar (PPBS) decreased significantly post-intervention in all experimental groups, underscoring the efficacy of the interventions (Figures 3-4). All reported p-values (mostly < 0.001) indicate robust and statistically significant changes, reinforcing the effectiveness of the intervention strategies (Table 4).

**Table 4:** Pre- and Post-intervention data for glycemic and psychological parameters are summarized Variables are presented as mean ± standard deviation; within-group differences were analyzed using paired t-test for normally distributed data and Wilcoxon Signed Rank Test (W) for non-normally distributed data, while between-group differences were analyzed using One-way ANOVA (F) or Kruskal–Wallis test (χ²) for non-normally distributed data, with post-hoc analysis performed using Tukey’s HSD or Dunn’s test with Bonferroni correction. HbA1c data were normally distributed in YG, GH, and CYG groups, while the Control group slightly deviated from normality; one-way ANOVA was applied because it is robust to mild deviations from normality, with Tukey’s HSD used for post-hoc comparisons; significance is indicated as *p < 0.05, **p < 0.01, and ***p < 0.001. HbA1c: glycated hemoglobin (%); FBS: fasting blood sugar (mg/dL); PPBS: postprandial blood sugar (mg/dL); PSS: perceived stress scale; DDS: diabetes distress scale; YG: yoga group; GH: gastro-hepatic pack; CYG: yoga + gastro-hepatic pack; W: Wilcoxon Signed Rank Test; MD: mean difference; HSD: Honestly Significant Difference

Variables	YG		GH		CYG		Control		p-value	Post-Hoc Pairwise Comparison	Between-Group Test
	Pre(Mean ± SD)	Post(Mean± SD)	Pre (Mean ± SD)	Post(Mean± SD)	Pre (Mean ± SD)	Post (Mean± SD)	Pre (Mean ± SD)	Post (Mean±SD)			
HbA1c	8.17±1.119	6.95±0.85***	8.04±0.098	6.93±0.67***	8.23±1.23	6.29±0.59***	8.12±0.93	7.46±1.03***^W^	p < 0.001	CYG < Control (MD = 1.17, p < 0.001) (Tukey’s HSD)	F = 16.18
FBS	144 ±28	102±6.25***^W^	136±10.7	122±12.07***	140.27±13.56	102.7±77.0***	139.5±12.4	134.7±17.1***^W^	p <0.001	CYG < GH (MD = 19.3, p < 0.001), CYG < Control (MD = 32.0, p < 0.001); YG < GH (MD = 20.0, p < 0.001) (Dunn’s test, Bonferroni)	χ²=63.67
PPBS	226±26.1	124±30.9***^W^	226±26.6	137±37.1***^W^	225±15.8	116±16.9***^W^	225±11.2	199±21.02***^W^	p < 0.001	CYG < GH (MD = 21.1, p < 0.001), CYG < Control (MD = 83.0, p < 0.001); YG < GH (MD = 13.0, p = 0.017), YG < Control (MD = 75.0, p < 0.001) (Dunn’s test, Bonferroni)	χ²=60.25
PSS	17.73±2.21	9.80±1.67***^W^	18.40±2.04	14.70±1.09***^W^	16.07±2.30	9.53±1.48***^W^	17.70±1.60	15.70±1.54***^W^	p < 0.001	YG < GH (MD = 5.42, p = 0.001), YG < CYG (MD = 6.18, p < 0.001); GH > Control (MD = 3.89, p = 0.003) (Dunn’s test, Bonferroni)	χ²=91.8
DDS	30.7±3.51	19.6±0.96***^W^	34.7±2.43	27.7±2.64***^W^	30.7±2.57	19.9±3.37***^W^	33±1.83	25.4±2.77***^W^	p < 0.001	YG < GH (MD 7.23, p<0.001), YG < CYG (MD 8.41, p<0.001), GH > Control (MD 4.67, p<0.001) (Dunn’s test, Bonferroni)	χ² =74.2

In the YG, mean HbA1c decreased from 8.17 %±1.12 to 6.95% ±0.85, in the GH from 8.04% ±0.98 to 6.93 %± 0.67, in the CYG from 8.23 %±1.23 to 6.29%±0.59, while the control group showed a marginal decrease from 8.12%±0.93 to 7.46%±1.03. Post-hoc analysis using Tukey’s honestly significant difference (HSD) revealed significantly lower post-intervention HbA1c levels in the CYG group compared to the Control group (MD = 1.17, p < 0.001). FBS levels demonstrated a reduction from 144 mg/dL±28 to 102 mg/dL ±6.25 in YG, GH from 136 mg/dL±10.7 to 122 mg/dL±12.07, CYG from 140.27 mg/dL± to 102.7 mg/dL±7.70, while the Control group showed only a mild reduction from 139.5 mg/dL ±12.4 to 134 mg/dL±17.1. Similarly, PPBS levels decreased from 226 mg/dL±26.1 to 124 mg/dL±30.9 in YG, 226 mg/dL±26.6 to 137 mg/dL±13.1 in GH, and CYG from 225 mg/dL± 15.8 to 116 mg/dL ±16.9, whereas the Control group showed a minimal reduction from 225 mg/dL±11.2 to 199 mg/dL±21.02. Dunn’s post-hoc test with Bonferroni correction demonstrated significantly lower FBS levels in the CYG group compared to the GH and Control groups, and significantly lower FBS levels in the YG group compared to the GH group. For PPBS, both CYG and YG groups showed significantly lower levels compared to the GH and Control groups (p < 0.05).

**Figure 5:**
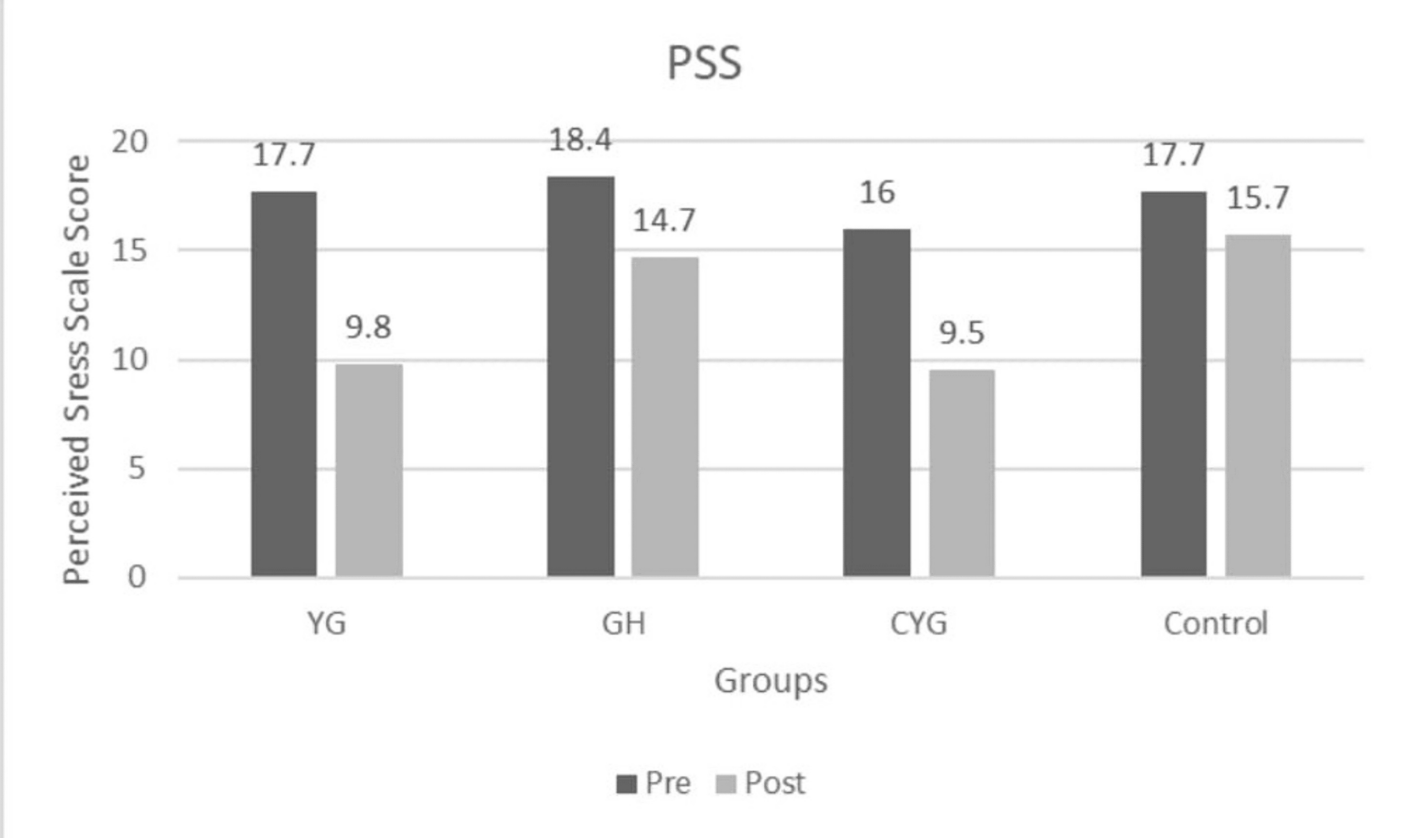
Graph 4: Perceived Stress Scale - Pre and Post Across Groups Pre and post intervention mean are shown for Yoga Group (YG), Gastro hepatic pack (GH), combined YG + GH, and Control groups. All intervention groups showed improvement, while the Control group showed a minimal change.

**Figure 6:**
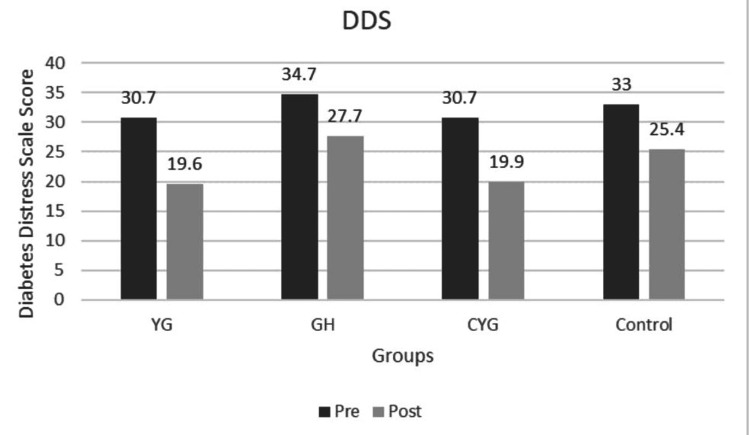
Graph 5: Diabetes Distress Scale - Pre and Post Across Groups Pre and post intervention mean are shown for Yoga Group (YG), Gastro hepatic pack (GH), combined YG + GH, and Control groups. All intervention groups showed improvement, while the Control group showed a minimal change.

PSS scores decreased significantly in the YG and CYG from baseline to post-intervention, with mean scores dropping from 17.73 to 9.80 and 16.07 to 9.53, respectively, both with p < 0.001 within groups (Figure 5). The GH showed a smaller reduction (18.40 to 14.70, p < 0.001), while the Control group had a minimal change (17.70 to 15.80, p < 0.001), indicating less effective stress reduction without structured intervention. The between-group p-value of < 0.001 confirms that the improvement in perceived stress was significantly greater in the intervention groups than in controls. Post-hoc analysis revealed that YG showed significantly greater reduction in PSS compared to GH (MD 5.42, p = 0.001) and CYG (MD 6.18, p < 0.001), and GH showed greater reduction than the Control group (MD 3.89, p = 0.003). The Cronbach’s alpha value of 0.62 indicates questionable internal consistency, suggesting that the scale has limited reliability and may require revision of its items. DDS scores, reflecting diabetes-related emotional distress, showed marked reductions in YG and CYG: from 30.7 to 19.6 and 30.7 to 19.9, respectively (p < 0.001), indicating substantial relief in distress (Figure 6). Post-hoc comparisons showed YG improved significantly more than GH (MD 7.23, p < 0.001) and CYG (MD 8.41, p < 0.001), and GH showed greater reduction than the Control group (MD 4.67, p < 0.001). The reliability analysis yielded a Cronbach’s alpha coefficient of 0.727, indicating acceptable internal consistency for the scale. The GH group also improved (34.7 to 27.7, p < 0.001), but less than YG and CYG, while the Control group showed the smallest decline (33.0 to 25.4, p < 0.001). The between-group p-value of < 0.001 indicates statistically significant superiority of the interventions over the control in reducing DD.

## Discussion

In this study, following 3 months of yoga and naturopathy intervention. In between the group analysis showed a significant reduction in HbA1c, FBS, PPBS, PSS, and DDS noted in three groups (p<0.001). In addition to biochemical improvements, the study revealed a significant decline in psychological stress indicators. Both PSS and DDS scores decreased markedly in the YG and CYG groups, with the latter showing the greatest benefit [21]. This finding supports earlier evidence that yoga reduces perceived stress, anxiety, and emotional distress by improving mindfulness, emotional regulation, and vagal tone. Reduced psychological stress could also have contributed indirectly to better glycemic outcomes, as chronic stress is a known precipitating factor for poor glycemic control through increased cortisol and catecholamine secretion. One study observed better glycemic control (HbA1c from 9.03 to 7.83%) after 40 days of yoga intervention [22]. In this study, HbA1c was significantly decreased from 8 to 6.12% in CYG. This result suggests that the intervention was successful in attaining good glycemic control because values in this range are typically thought to be representative of the best possible management of diabetes. Better long-term glucose homeostasis and better metabolic regulation among participants in the combined group are suggested by the observed decrease.

A study followed for 40 days of yoga has observed a reduction of FBG (from 210.7 to 140.4 mg/dL) and PPBG (from 305.5 to 230.5 mg/dL)[23]. Another study followed for the same period of time showed a significant reduction of FBG (190.1-141.5 mg/dL) and PPBG (276.5-201.7 mg/dL) [24]. This study reinforces the findings of the present research, which showed a reduction in FBS from 140 to 102mg/dL and PPBS (225 to 120mg/dL) after three months of yoga practice. The majority of yoga treatments significantly reduced the HbA1c level in T2DM patients. Yoga has proven to be effective in reducing depression and stress, improving cognitive function, and enhancing mindfulness, thus making it a complementary approach in the management of diabetes [25].

Yoga has a pair of benefits: it reduces stress and inhibits the activity of the sympathetic nervous system, which assists in avoiding diseases linked to stress and lower blood sugar. In addition to improving circulation and oxygen delivery to essential organs, the combination of SKY’s stretching exercises, asanas, and controlled breathing reduces reliance on anaerobic metabolism, improves circulation, and enhances oxygen delivery [26]. During SKY, guided meditation lowers oxidative stress, anxiety, and stress perception, improving general well-being. The induction of a relaxing state is suggested by the decrease in serum lactate levels following SKY exercises [27-29].

The perception that diabetes is consuming excessive amounts of mental and physical energy on a daily basis and the fear of developing major long-term problems were the two primary emotions that contributed to high emotional Diabetes Distress [30]. Increased parasympathetic drive, calming of the stress response systems, including increased production of hormones like prolactin, oxytocin, and vasopressin, decreasing production of cortisol and adrenocorticotropin hormone, and normalization of serum brain-derived neurotrophic factor level are some of the theories put forth by studies to understand the mechanism by which SKY contributes to the state of calm alertness [31].

Patients with type 2 diabetes who had a 20-minute GHP treatment showed a significant decrease in blood glucose levels both immediately and after 15 minutes, according to a pilot group pre - post trial [32]. In our study, a significant improvement was observed in gastro-hepatic packing (GH), which reduced FBS from 151 to 127 mg/dL and PPBS from 226 to 143 mg/dL, indicating better glycemic control among participants. This reduction highlights the potential effectiveness of the intervention in managing blood glucose levels in type 2 diabetes.

Both hot- and cold-water therapies are used in the GHP. A fomentation bag is used for the hot intervention, and an ice bag is used for the cold intervention. Specific abdominal regions, especially the liver and pancreas, are the focus of GH pack therapy. Hot applications typically cause the skin's blood vessels to dilate, which increases the activity and metabolism of the skin and underlying tissues. This may lead to an increase in the skin's and the muscles' utilization of glucose. On the other hand, cold application results in the contraction of cutaneous blood vessels, which lowers the temperature of the afflicted areas and decreases skin activity. In the deeper vascular system, this cold exposure may result in compensatory vasodilatation, which would increase blood flow to the tissues underneath the exposed area. Because of this, higher blood flow can raise metabolic rate and aid in preserving a steady deep tissue temperature [33]. High levels of perceived stress in Type II DM elevate cortisol levels, which can worsen insulin resistance and blood glucose. Chronic stress exacerbates self-management practices, such as food, exercise, and medication adherence, and has a role in the onset and progression of diabetes. The mental strain and anxiety that are directly associated with controlling diabetes is known as DD. Longer disease duration, increased insulin resistance, and worse glycemic control have all been associated with elevated diabetic distress. Diabetes patients who are distressed frequently experience frustration, burnout, or a lack of support, which lowers their motivation and makes them less successful. Glycemic control is the primary indicator of therapy success in the majority of type II diabetes studies; however, this study stands out for concentrating on both physiological and psychological factors at the same time, offering a more comprehensive evaluation of patient outcomes.

There are a number of restrictions on the study. First, the results may not be as broadly applicable as they may be due to the limited sample size (n = 120). More reliable evidence would come from a multicentric study with a larger sample. Second, there was little consistency of yoga instruction; individuals might have received instruction of differing quality, which could have an impact on the results. Third, the results may have been impacted by confounding variables that were not controlled, such as concurrent medications, dietary practices, and lifestyle choices. Fourth, a thorough assessment of metabolic health was limited since metabolic measures such as lipid profiles, liver function tests, and renal function tests were not evaluated. Lastly, mechanistic interpretation is further limited by the comparatively short follow-up duration and lack of sophisticated biomarkers or imaging evaluations.

Future research should address these aspects to better explore the mechanistic pathways - such as hormonal, neuroendocrine, and inflammatory markers - through which these interventions exert their metabolic and psychological effects.

## Conclusions

Integrating yoga and naturopathic treatments into standard diabetes treatment provides a secure, efficient, and comprehensive approach to treating the psychological and physiological effects of type 2 diabetes. In addition to improving glycemic control, these therapies help reduce stress and emotional strain, which raises quality of life in general. Future extensive, long-term research is necessary to assess how sustainable these advantages are and how they affect complications associated with diabetes.

## Appendices

The Diabetes Distress Scale (DDS-17) and Perceived Stress Scale (PSS-10) were included in the research. The authors converted the questionnaires into tables for administration and included them as appendices. The publications from the original scale have been cited.

Appendix A

**Table 5:** Perceived Stress Scale (PSS-10)

In the last month, how often have you...	Never	Almost Never	Sometimes	Fairly Often	Very Often
1. Been upset because of something that happened unexpectedly?	0	1	2	3	4
2. Felt that you were unable to control the important things in your life?	0	1	2	3	4
3. Felt nervous and “stressed”?	0	1	2	3	4
4. Felt confident about your ability to handle your personal problems?	0	1	2	3	4
5. Felt that things were going your way?	0	1	2	3	4
6. Found that you could not cope with all the things that you had to do?	0	1	2	2	4
7. Been able to control irritations in your life?	0	1	2	3	4
8. Felt that you were on top of things?	0	1	2	3	4
9. Been angered because of things that were outside of your control?	0	1	2	3	4
10. Felt difficulties were pulling up so high that you could not overcome them?	0	1	2	3	4

Appendix B

**Table 6:** Diabetes Distress Scale (DDS-17)

During the Past month	Not a problem	A Slight Problem	A Moderate Problem	Somewhat Serious Problem	A Serious Problem	A very Serious Problem
Feeling that diabetes is taking up too much of my mental and physical energy everyday.	1	2	3	4	5	6
Feeling that my doctor doesn’t know enough about diabetes and diabetes care.	1	2	3	4	5	6
Feeling angry, sacred, and/or depressed when I think about living with diabetes.	1	2	3	4	5	6
Feeling that my doctor doesn’t give me clear enough directions on how to manage my diabetes.	1	2	3	4	5	6
Feeling that I am not testing my blood sugars frequently enough.	1	2	3	4	5	6
Feeling that I am often failing with my diabetes routine.	1	2	3	4	5	6
Feeling that friends or family are not supportive enough of self-care efforts (e.g planning activities that conflict with my schedule, encouraging me to eat the “wrong” foods).	1	2	3	4	5	6
Feeling that diabetes controls my life.	1	2	3	4	5	6
Feeling that my doctor doesn’t take my concerns seriously enough	1	2	3	4	5	6
Not feeling confident in my day-to-day ability to manage diabetes.	1	2	3	4	5	6
Feeling that I will end up with serious long-term complications, no matter what I do.	1	2	3	4	5	6
Feeling that I am not sticking closely enough to a good meal plan.	1	2	3	4	5	6
Feeling that friends or family don’t appreciate how difficult living diabetes can be.	1	2	3	4	5	6
Feeling overwhelmed by the demands of living with diabetes.	1	2	3	4	5	6
Feeling that I don’t have a doctor who I can see regularly enough about my diabetes.	1	2	3	4	5	6
Not feeling motivated to keep up my diabetes self management.	1	2	3	4	5	6
Feeling that friends or family don’t give me the emotional support that I would like.	1	2	3	4	5	6
